# Mr. Syer, on Monstrosity

**Published:** 1801-02

**Authors:** John Syer

**Affiliations:** Cowbridge, South Wales.


### *To the Editors of the Medical and Phyſical Journal.*

GENTLEMEN,

I Have not been inattentive to the utility or celebrity of your Medical Journal; and although I have no reliſh for controverſy in general, yet, a Caſe of Monſtroſity recorded in your 19th Number, by Mr. Simmons, appears highly curious, and deſerving of further conſideration. Every one who contemplates the endleſs varieties of animated Nature but with the ſlighteſt attention, muſt admit of the aſtoniſhing wiſdom with which all beings, but particularly thoſe endowed with intelligence, are adapted to the different modes of exiſtence for which Providence has deſigned them. The philoſopher muſt frequently lament, that aided by the profoundeſt accuracy and perſeverance, he can yet aſcend but ſo few ſteps in diſcovering the eſſential operations of mind on matter: Could we once diſpel the the cloud that obfcures our faculties on this point, phyfiology would acquire fomething like demonftrative truth. Although the prefent age is particularly diftinguifhed by its thirft for experiment, and a fcrupulous adherence to facts on moft fubjects of fcience, yet, a further defideratum is no lefs neceffary; namely, caution in generalizing our ideas. We muft keep in view this axiom, that "poft hoc, ergó propter hoc," is not always in phyfiology a legitimate conclufion. I fhall briefly notice a few particulars in the cafe in queftion, and then take the liberty of fuggefting fuch ideas as my own confideration of it has furnifhed me with, with due deference, however, to the author of the paper, who is certainly entitled to the thanks of your readers for the communication. I beg leave to premife one or two obfervations in this place, and particularly, that cafes of fuch a fingular defcription as the prefent, fo far from removing the difficulties that occur with refpect to the illuftration of the phyfiology of the brain and nerves, rather indicate the fimple provifions which Nature can employ in continuing the exiftence of the human fœtus. The author's mode of reafoning in the inftance before us, is not unlike that of a natural philofopher, who would ground the fundamental fyftem of the laws of motion on the eccentricities of a comet. I will juft quote a paffage from Steno, which the immortal Winflow has fubjoined to his Anatomy of the brain:—"The foul which imagines it can penetrate into every thing without it, and that nothing can fet bounds to its knowledge, is neverthelefs utterly at a lofs to defcribe its own habitation, and is no where more to feek than at home."—Mr. S. obferves, that there was nothing preternatural in the ftructure or relative fituation of the thoracic and abdominal vifcera, and that they were all fupplied in the ufual manner with their proper nerves. He fays, below the occiput there was a confiderable difplaced portion of vertebra, both cervical and dorfal, and in thefe parts he found no veftige of medullary matter; confequently, I am at a lofs to conceive how he difcovered the origin of the phrenc. nerve, and the origination of the axillary plexus; for he ftates, that the upper extremities were furnifhed with their proper nerves. I am induced to hazard a conjecture, that the above cafe was a morbid one in utero, and that it was not originally conftituted in the fituation it prefented itfelf in at the birth; particularly, as I am ready to agree with the author in part, that the connection between the brain and circulation of the blood in the fœtal ftate, is not fo obvioufly neceffary. It may not be very remote from the truth, to confider it as a malformation, produced by partial abforption; this, however, is merely offered as a query. I am, however, much againft the author's general

 doctrine, doctrine, which he has thought fo clearly deducible from this inftance: Firft, that nervous influence is not at all neceffary in the evolution of the fœtus; indeed, it is anfwered by the univerfal exiftence of a brain and nerves in the fœtus; and it is well known, that the head is one of the firft parts of the ftructure of every animal that manifefts itfelf after conception; and therefore, we are furely not to refufe our belief in the importance of this fyftem, merely from a folitary exception. The fecond pofition, viz. "that the fœtus in utero does not poffefs fenfation," is not lefs liable to animadverfion, fince, from the different ftages of its evolution, Nature has appeared to have intended, that the brain in particular fhould arrive very flowly at perfection; between the extremes of fimple fenfation and refinement of intellect, the links muft furely be very remote. It is perfectly confonant to the foundeft wifdom to believe, that a part which was to be the centre of all our thoughts and affections, fhould receive acceffions of phyfical ftrength in proportion to the neceffary demands of the animal; if fo, it is more reafonable to hazard an opinion, (which, however, can only reft on that bafis) that the brain and nerves furnifhed an important part in the human frame ab origine; and that fo far from being a work of fupererogation at this period, ought we not to conceive it as an additional argument in proof of the unerring rules of Providence, difplayed alike in every part of the animal creation? Although I have ventured to regard this cafe as denoting partial difeafe, ftill, the healthy appearance of the child otherwife, may be thought to weaken this fuppofition; neverthelefs, the author's conclufions are equally liable to our exception. The duplicature of membrane within the cranium and fpinal canal, would feem to favour the opinion of the primary exiftence of medullary fubftance, fince fuch a protection to thefe parts would feem, a priori, quite fuperfluous. With regard to the functions of refpiration and fenfation being coeval, it does not appear ftrictly neceffary, fince the former, in many inftances, is perfectly involuntay, and in this refpect, coincides with the œconomy of the heart. In the cafe before us, one thing is worthy of obfervation, that, notwithftanding the want of cerebrum and cerebellum, yet we find that the nerves were all within their ufual courfe, (except the optic and recurrents, which were not included in the defcription) as if they imparted fome influence, independant of the grand fource of fenfation; this idea is curforily intimated by the ingenious Dr. Baillie in his Morbid Anatomy. Before I conclude, I fhall juft obferve, that I have feen an inftance of an arterial fyftem without a heart, in a fmall fœtus, at about the fifth month; the aorta with its curvature, fupplied its place. Whatever analogy logy may be drawn from this fact, I ſhall forbear to point out, ſince truth, not diſputation, is the object of my reſearches.—Thus, gentlemen, I have preſumed to offer a few reflections, which might be uſefully extended on ſo intereſting, but abſtruſe, a branch of phyſiology as the preſent; and if they ſhould accord with the ſpirit of your very valuable Journal, you are at full liberty to inſert them. I am, &c.

*Cowbridge, South Wales.* JOHN SYER.

